# Diagnostic Performance of Ultrasonography Versus Magnetic Resonance Cholangiopancreatography in Biliary Obstruction

**DOI:** 10.7759/cureus.33915

**Published:** 2023-01-18

**Authors:** Sunny Swaraj, Manoranjan Mohapatra, Gitanjali Sathpathy, Rajesh Yalamanchi, Kamal Sen, Sreedhar M Menon, Ajeet Madhesia, Suma M Kumaraswamy, Kolluru Radha Krishna, Devesh V Bobde

**Affiliations:** 1 Radiodiagnosis, Kalinga Institute of Medical Sciences, Bhubaneswar, IND

**Keywords:** imaging, ultrasonography, endoscopic retrograde cholangiopancreatography, magnetic resonance cholangiopancreatography, mri, ercp, biliary obstruction, mrcp, usg

## Abstract

Background

In a suspected case of biliary obstruction with clinical and laboratory data suggesting obstructive jaundice, the major goal is to confirm the presence of obstruction, its nature and cause, location, and extent. Ultrasonography (USG) and magnetic resonance cholangiopancreatography (MRCP) are primarily used to diagnose suspected biliary tract illnesses. The aim of the study is to evaluate and compare the accuracy of MRCP and USG with endoscopic retrograde cholangiopancreatography (ERCP)/surgical/histopathological outcomes for finding the cause and level of obstruction in the case of clinically suspected biliary obstruction.

Methods

This was a prospective observational study conducted at Kalinga Institute of Medical Sciences and Pradyumna Bal Memorial Hospital, Bhubaneswar, India, from September 2020 to September 2022 on 120 patients. It included patients with clinical suspicion of biliary obstruction who underwent both USG and MRCP. Characteristics of the obstruction were evaluated for both benign and malignant lesions through USG and MRCP. The findings were then correlated with ERCP, histopathology, or surgery to calculate the diagnostic performance of the former two modalities.

Results

Out of 120 patients, USG was correctly able to predict the cause of obstruction in 40 patients. The sensitivity, specificity, and accuracy of detecting the nature of obstruction by USG were 33.3%, 84%, and 48.9%, respectively. The overall diagnostic accuracy of USG in predicting the site of obstruction was 64.3%. MRCP was correctly able to predict the cause of obstruction in 113 patients. The sensitivity, specificity, and accuracy of detecting the nature of obstruction by MRCP were 94.1%, 91.9%, and 94.8% respectively. The overall diagnostic accuracy of MRCP in predicting the site of obstruction was 98.33%. Out of 120 patients, no cause of biliary obstruction could be found in 71 patients by USG, out of which the correct diagnosis was made in 67 patients through MRCP.

Conclusion

USG should be used as the initial screening modality of choice for predicting the level and nature of obstruction in patients with a clinical suspicion of obstructive jaundice. MRCP should be the radiological investigation of choice in patients with clinical suspicion of obstructive jaundice. MRCP has the potential to become the new "Gold standard" investigation for diagnosis in patients with biliary obstruction owing to its excellent diagnostic performance, and non-invasiveness.

## Introduction

Obstructive jaundice is a prevalent clinical issue. In a suspected case of biliary obstruction with clinical and laboratory data suggesting obstructive jaundice, the major goal is to confirm the presence of obstruction, its nature and cause, location, and extent. Primarily, ultrasonography (USG) and magnetic resonance cholangiopancreatography (MRCP) are used to diagnose biliary tract illnesses.

Due to its many benefits, such as its simplicity of use, low cost, and lack of ionizing radiation, USG is used as the initial screening tool. The visualization of the distal common bile duct (CBD) and pancreas is limited in around 30% of instances due to obscuration caused by bowel gas [1,2]. MRCP, unlike USG, is not affected by bowel gas and gives a clear view of the hepatobiliary system [3]. It has inherent high contrast resolution, the ability to completely map the biliary ductal system, no need for contrast media, multiplanar capability, and virtually artifact-free display of anatomy and pathology in patients with biliary obstruction [4]. The aim of our study is to evaluate and compare the diagnostic performance of USG and MRCP with ERCP/surgical/histopathological outcomes for finding the cause and level of obstruction in the case of clinically suspected biliary obstruction.

## Materials and methods

Study criteria

The study was a prospective observational study conducted at Kalinga Institute of Medical Sciences and Pradyumna Bal Memorial Hospital, Bhubaneswar, India, from September 2020 to September 2022. Patients with clinical suspicion of biliary obstruction who underwent both USG and MRCP were included in the study. Those patients who either went for only USG or MRCP were not included in the study.

Machines

Transabdominal USG was performed with Voluson™ S10 (GE HealthCare Technologies Inc., Chicago, Illinois, United States) and Affiniti 30 (Koninklijke Philips N.V., Amsterdam, Netherlands) machines using a curvilinear probe of 2-5 MHz frequency. MRCP was performed with a GE Signa HDxT 1.5 Tesla MRI machine using T1WI, coronal and axial T2 single-shot fast spin echo (SSFSE) pulse sequence, apparent diffusion coefficient (ADC), diffusion-weighted imaging (DWI), coronal and axial fast imaging employing steady-state acquisition (FIESTA), axial and coronal three-dimensional MRCP, and thick slab MRCP sequences.

The nature and level of obstruction were evaluated for both benign and malignant lesions through both modalities. The findings were then correlated with ERCP, histopathology, or surgery to calculate the diagnostic performance of USG and MRCP.

## Results

**Figure 1:**
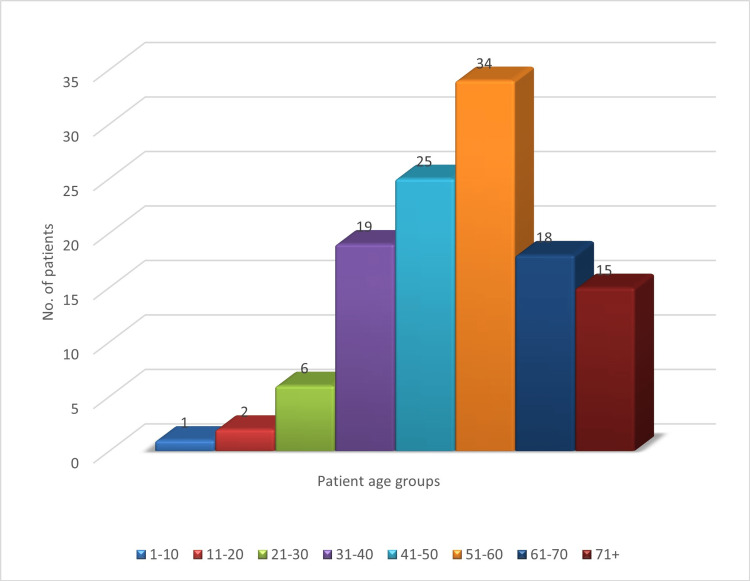
Age distribution of the population

During this two-year period, of the 120 patients who were referred for both USG and MRI, 57 (47.5%) were male and 63 (52.5%) were female, showing a slight female preponderance. The youngest patient was one year old and the oldest was 88 years old. The mean age of patients was 52.2 years (Figure 1).

**Figure 2:**
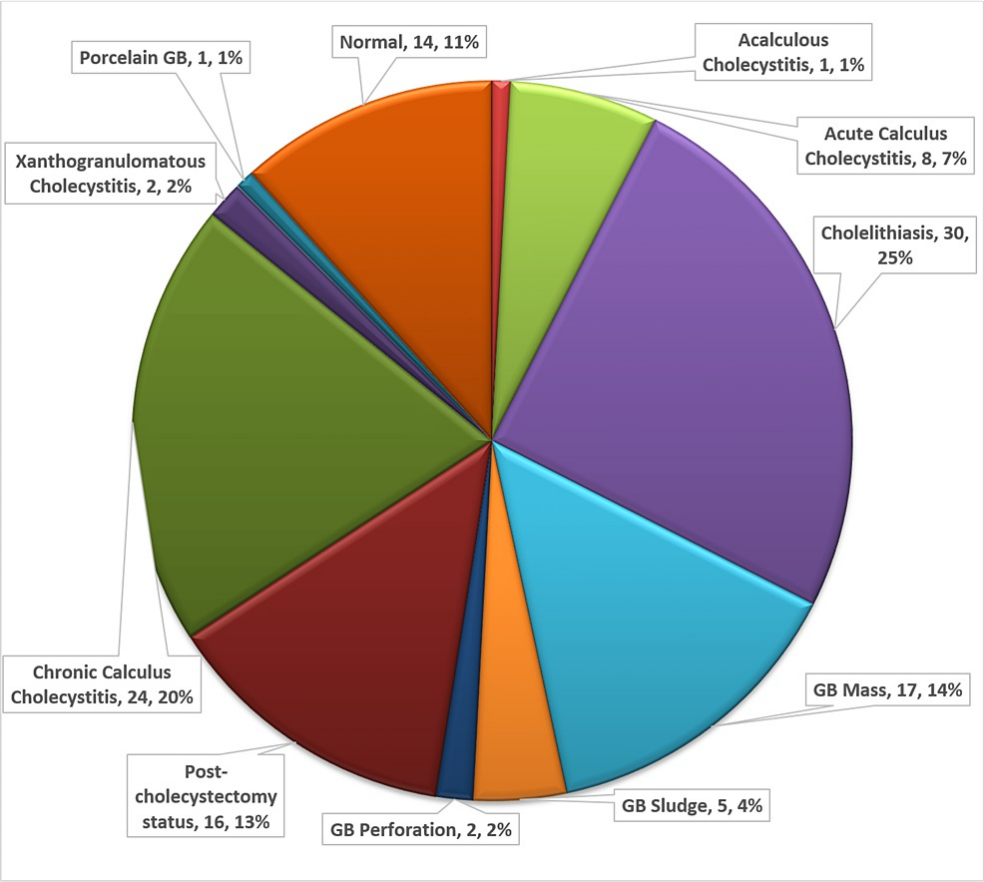
Gallbladder status in the patients GB: gallbladder

Thirty patients (25%) had cholelithiasis without any signs of cholecystitis. Twenty-four patients (20%) had chronic calculus cholecystitis and eight patients (6.67%) had acute calculus cholecystitis. Gallbladder cancer was found in 17 patients (14.17%). Post-cholecystectomy status was noted in 16 patients (13.3%). No pathology was found in 14 patients (11.67%) (Figure 2).

**Figure 3:**
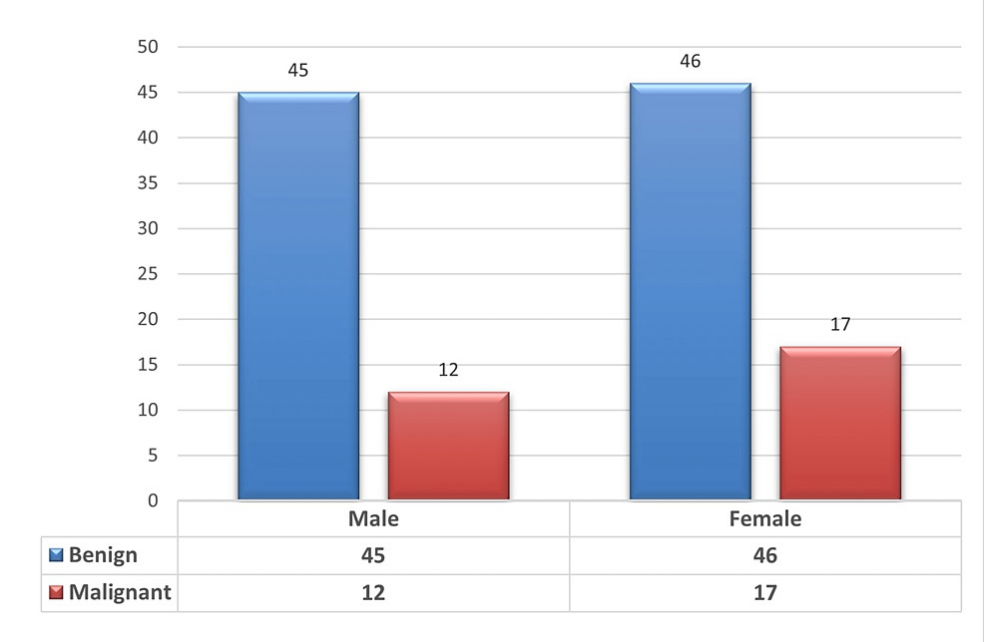
Demographic characteristics of nature of lesions

**Figure 4:**
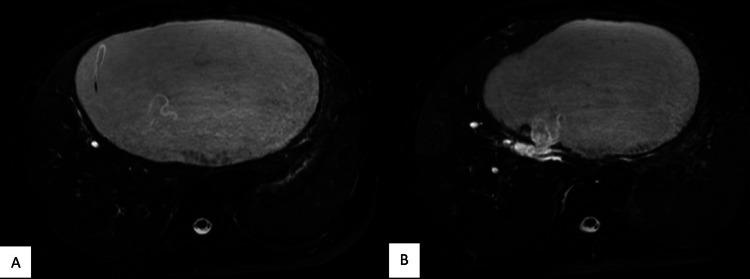
Intrahepatic hydatid cyst. (A) Axial three-dimensional reconstructed image shows serpentine-like floating structures, internal floating membranes; (B) Axial three-dimensional reconstructed image shows cysto-biliary communication through left hepatic duct

In our study, benign pathology was found in 91 patients (75.8%), whereas malignant pathology was found in 29 patients (24.2%) (Figure 3). In benign lesions, 67 cases (55.8%) of choledocholithiasis were reported, out of which four were associated with other pathologies (one with carcinoma pancreas, one with a blocked self-expanding metallic stent (SEMS), and two with Mirizzi syndrome). Twelve cases (10%) of benign biliary stricture and five cases (4.1%) of Mirizzi syndrome were reported. Two cases (1.67%) each of intrahepatic hydatid cysts (Figure 4), ampullary stenosis, and periampullary diverticulum causing biliary obstruction were reported. In our study, the most common pathology leading to obstructive jaundice was choledocholithiasis. In malignant lesions, 17 cases (14.17%) of gallbladder carcinoma, followed by eight cases (6.67%) of cholangiocarcinoma and two cases (1.67% each) of pancreatic carcinoma and periampullary carcinoma were reported (Table 1).

**Table 1:** Total case distribution

1.	Malignant	Periampullary Carcinoma	2
2.		Carcinoma Pancreas	2
3.		Gallbladder Carcinoma	17
4.		Cholangiocarcinoma	8
5.	Benign	Ampullary Stenosis	2
6.		Autoimmune Hepatitis	1
7.		Benign Biliary Stricture	12
8.		Biliary Sludge	1
9.		Choledocholithiasis	63
10.		Hydatid Cyst	2
11.		Mirizzi Syndrome	5
12.		Main Pancreatic Duct Stricture	1
13.		Periampullary Diverticulum	2
14.		Type IV A Choledochal Cyst	1
15		Blocked Self-Expanding Metallic Stent with Choledocholithiasis	1

Diagnostic performance of USG versus MRCP in benign lesions

Out of 91 patients presenting with a benign pathology, USG was able to correctly diagnose the cause in 19 patients (20.9%). In 66 patients (72.5%), no obstructive cause could be found. In three patients, Mirizzi syndrome was erroneously diagnosed as choledocholithiasis. A benign stricture of the left hepatic duct was missed in one patient post hepaticojejunostomy. In another patient, a benign distal CBD stricture was erroneously diagnosed as a type-1 choledochal cyst. A patient with a blocked self-expandable metallic stent in the CBD with features of biliary obstruction and porcelain gallbladder was erroneously suspected of having gallbladder carcinoma. The sensitivity, specificity, and accuracy in detecting benign lesions by USG were 20.9%, 79.3%, and 35% respectively.

**Figure 5:**
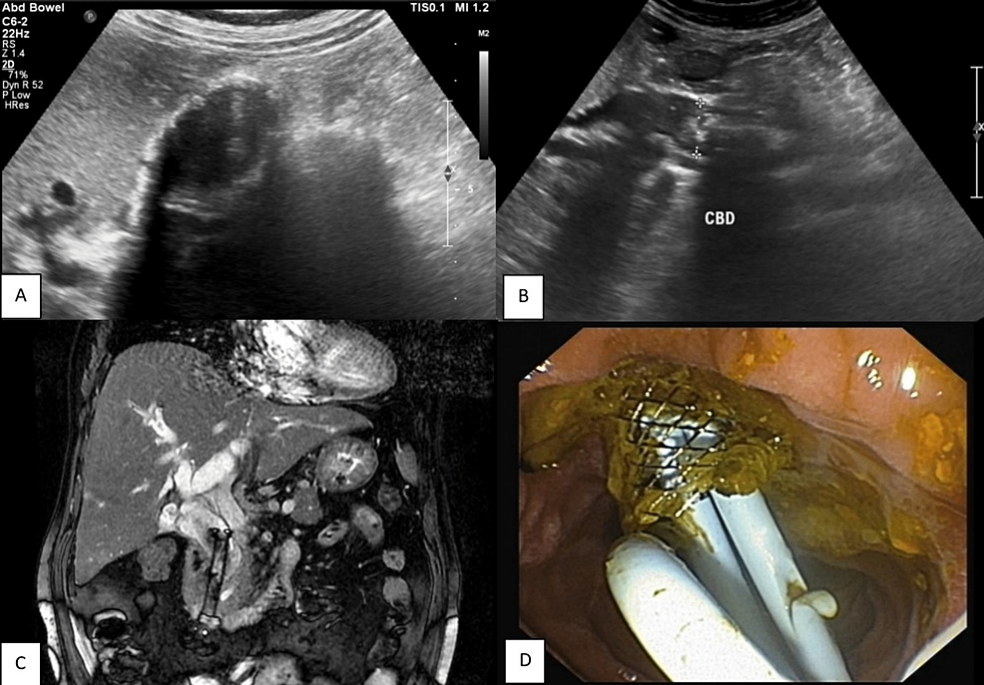
Blocked self-expanding metallic stent with porcelain GB. (A) Transabdominal ultrasonography shows calcified and thickened gallbladder wall; (B) Transabdominal USG shows dilated common bile duct with stent in situ and non-homogenous echogenic material; (C) Coronal T2 SSFSE shows cholelithiasis with porcelain GB and bilobar intrahepatic biliary radicle dilatation with common bile duct stent in situ; (D) Removal of blocked stent GB: gallbladder; SSFSE: single-shot fast spin echo

**Figure 6:**
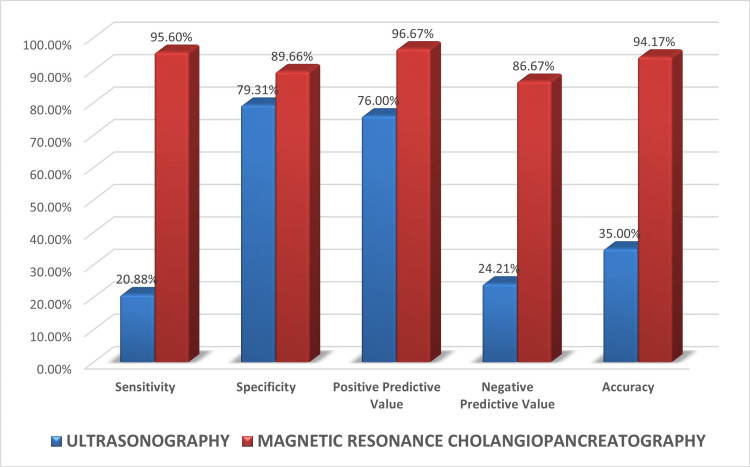
Diagnostic performance of USG and MRCP in estimating cause of obstruction in benign lesions MRCP: magnetic resonance cholangiopancreatography

Out of 91 patients presenting with a benign pathology, MRCP was able to correctly diagnose the cause in 87 patients (95.6%). One case of ampullary stricture was erroneously diagnosed as choledocholithiasis, and another case of choledocholithiasis was misdiagnosed as Mirizzi syndrome. Another case of blocked SEMS and choledocholithiasis with porcelain gallbladder was suspected to be carcinoma gallbladder (Figure 5). No cause of obstruction could be detected in one case, which was later found to be a small calculus in the ampullary region. The sensitivity, specificity, and accuracy in detecting benign lesions by MRCP were 95.6%, 89.6%, and 94.2%, respectively (Figure 6).

Out of 120 patients, 67 were reported to have been associated with choledocholithiasis. In 53 patients, acalculous pathologies were reported. The sensitivity, specificity, and accuracy of detecting acalculous pathology by USG were 54.72%, 79.10%, and 68.33%, respectively. Out of 67 cases of choledocholithiasis, only 15 (23.4%) were diagnosed on USG whereas 65 cases (97%) were diagnosed on MRCP. The sensitivity, specificity, and diagnostic accuracy of USG and MRCP for choledocholithiasis were 22%, 92.4%, 53.3% and 97%, 98.1%, 97.5%, respectively. 

**Figure 7:**
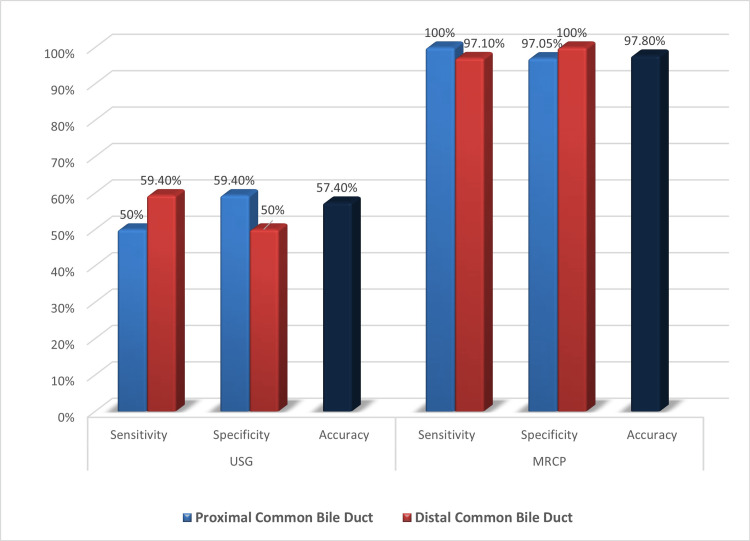
Diagnostic performance of USG and MRCP in estimating level of obstruction in benign lesions MRCP: magnetic resonance cholangiopancreatography

Out of 22 patients with proximal CBD obstruction, USG was correctly able to predict the level of obstruction in 11 patients (sensitivity: 50%) and MRCP was correctly able to predict the level of obstruction in all patients (sensitivity: 100%). Out of 69 patients with distal CBD obstruction, USG was correctly able to predict the level of obstruction in 41 patients (sensitivity: 59.4%) and MRCP was correctly able to predict the level of obstruction in 67 patients (sensitivity: 97.1%). The diagnostic accuracy in predicting the level of obstruction for USG and MRCP came out to be 57.4% and 97.8%, respectively (Figure 7, Table 2).

**Table 2:** Level of obstruction in benign lesions MRCP: magnetic resonance cholangiopancreatography; ERCP: endoscopic retrograde cholangiopancreatography

		Histopathology/ERCP/Surgery	
		Proximal common bile duct	Distal common bile duct
USG	Proximal common bile duct	11	0
	Inconclusive	8	28
	Distal common bile duct	3	41
		Proximal common bile duct	Distal common bile duct
MRCP	Proximal common bile duct	22	1
	Inconclusive	0	1
	Distal common bile duct	0	67

Diagnostic performance of USG versus MRCP in malignant lesions

Out of 29 patients presenting with a malignant pathology, USG was able to correctly diagnose the cause in 21 patients (72.5%). In five (17.2%) patients, no cause of obstruction could be determined. Two patients were diagnosed with choledocholithiasis, which turned out to be gallbladder carcinoma with distal CBD mass and periampullary infiltrating carcinoma. One patient with gallbladder carcinoma was erroneously diagnosed with cholangiocarcinoma. The sensitivity, specificity, and accuracy in detecting malignant lesions by USG were 72.4%, 98.9%, and 92.5%, respectively.

**Figure 8:**
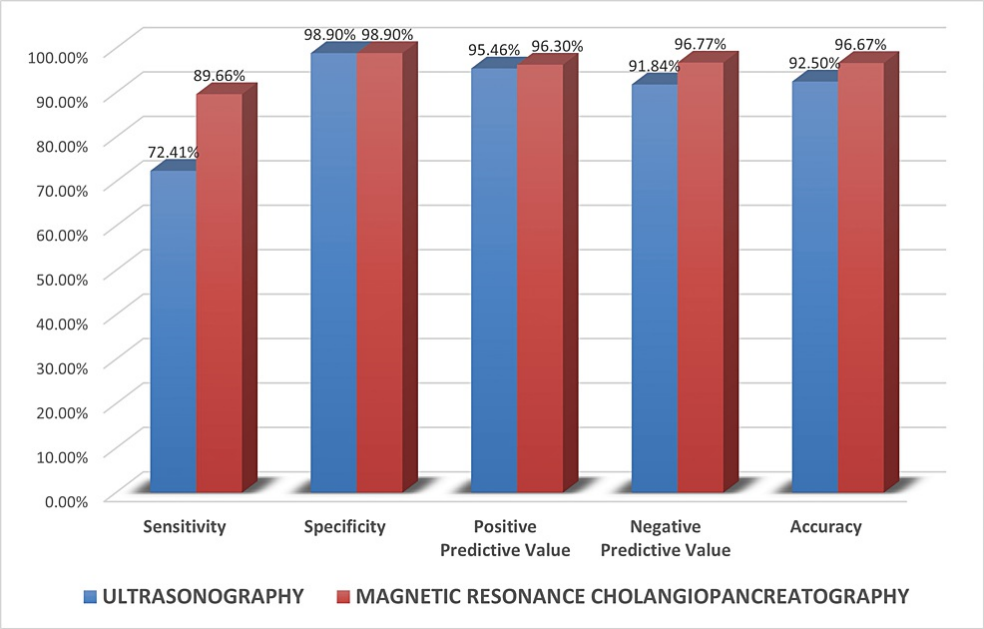
Diagnostic performance of USG and MRCP in estimating cause of obstruction in malignant lesions MRCP: magnetic resonance cholangiopancreatography

Out of 29 patients presenting with a malignant pathology, MRCP was able to correctly diagnose the cause in 26 patients (89.6%). One case of periampullary carcinoma and one of pancreatic carcinoma were each diagnosed erroneously as benign distal CBD strictures. One case of cholangiocarcinoma was erroneously suspected to be Mirizzi syndrome with CHD narrowing. The sensitivity, specificity, and accuracy in detecting malignant lesions by MRCP were 89.6%, 98.9%, and 96.7% respectively (Figure 8).

**Figure 9:**
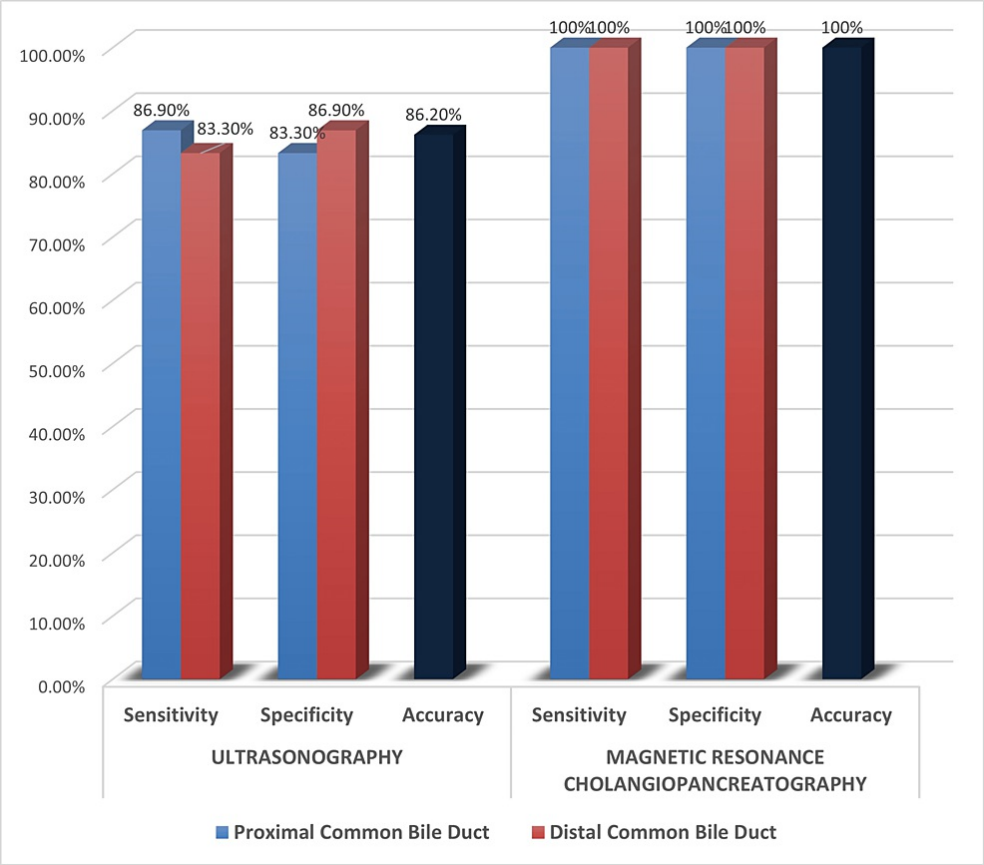
Diagnostic performance of USG and MRCP in estimating level of obstruction in malignant lesions MRCP: magnetic resonance cholangiopancreatography

Out of 23 patients with proximal CBD obstruction, USG was correctly able to predict the level of obstruction in 20 patients (sensitivity: 86.9%). Out of six patients with distal CBD obstruction, USG was correctly able to predict the level of obstruction in five patients (sensitivity: 83.3%). MRCP was correctly able to predict the level of obstruction in both proximal and distal CBD obstruction patients (sensitivity: 100%). The diagnostic accuracy in predicting the level of the malignant lesion for USG and MRCP were 86.20% and 100% respectively (Figure 9, Table 3).

**Table 3:** Level of obstruction in malignant lesions MRCP: magnetic resonance cholangiopancreatography; ERCP: endoscopic retrograde cholangiopancreatography

		Histopathology/ERCP/Surgery	
		Proximal common bile duct	Distal common bile duct
USG	Proximal common bile duct	20	1
	Inconclusive	2	0
	Distal common bile duct	1	5
		Proximal common bile duct	Distal common bile duct
MRCP	Proximal common bile duct	23	0
	Distal common bile duct	0	6

Overall diagnostic performance

Out of 120 patients, USG was correctly able to predict the cause of obstruction in 40 patients. The sensitivity, specificity, and accuracy of detecting the nature of obstruction by USG were 33.3%, 84%, and 48.9% respectively. The overall diagnostic accuracy of USG in predicting the level of obstruction was 64.3%.

**Figure 10:**
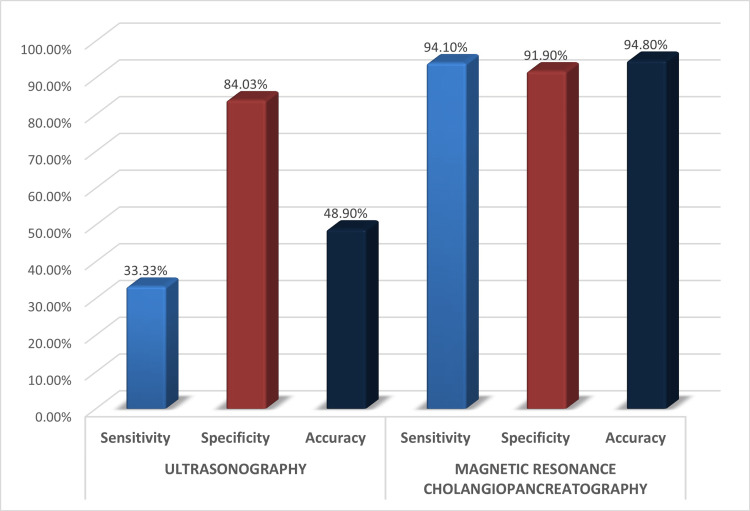
Diagnostic performance of USG versus MRCP in predicting cause of obstruction in all 120 patients MRCP: magnetic resonance cholangiopancreatography

**Figure 11:**
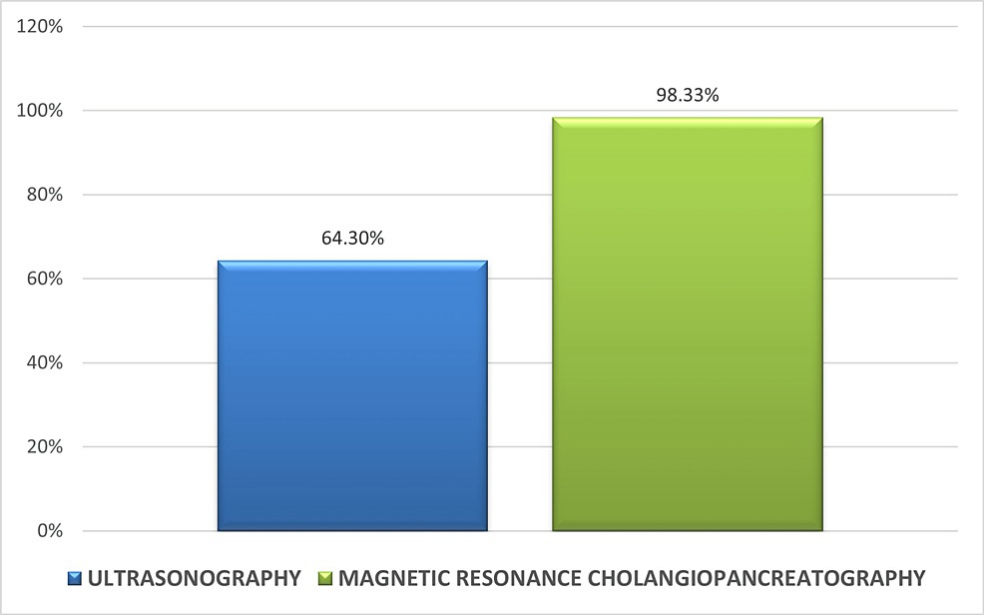
Diagnostic accuracy of USG versus MRCP in predicting level of obstruction MRCP: magnetic resonance cholangiopancreatography

Out of 120 patients, MRCP was correctly able to predict the cause of obstruction in 113 patients. The sensitivity, specificity, and accuracy of detecting the nature of obstruction by MRCP were 94.1%, 91.9%, and 94.8% respectively. The overall diagnostic accuracy of MRCP in predicting the level of obstruction was 98.33% (Figures 10, 11).

Out of 120 patients, no cause of biliary obstruction could be found in 71 patients by USG, out of which the correct diagnosis was made in 67 patients through MRCP. The sensitivity, specificity, and diagnostic accuracy of MRCP in detecting the cause of obstruction in inconclusive USG for biliary obstruction were 94.37%, 93.88%, and 94.17%, respectively. The diagnostic performance of USG and MRCP for individual lesions are shown in Table 4.

**Table 4:** Diagnostic performance of USG and MRCP in biliary obstructive lesions MRCP: magnetic resonance cholangiopancreatography

		USG			MRCP		
		Sensitivity	Specificity	Accuracy	Sensitivity	Specificity	Accuracy
1	Ampullary Stenosis	50	-	99	50	-	99
2	Autoimmune Hepatitis	0	-	99	100	-	100
3	Benign Biliary Stricture	17	100	92	100	99	99
4	Biliary Sludge	0	99	98	100	100	100
5	Choledocholithiasis	22	92	53	97	98	97
6	Carcinoma Pancreas	50	100	99	100	100	100
7	Gallbladder Carcinoma	76	99	95	100	99	99
8	Cholangiocarcinoma	87	99	98	87	100	99
9	Hydatid Cyst	100	100	100	100	100	100
10	Mirrizi Syndrome	20	100	97	100	98	98
11	Main Pancreatic Duct Stricture	100	100	100	100	100	100
12	Periampullary Carcinoma	0	100	98	50	100	98
13	Periampullary Diverticulum	0	100	98	100	100	100
14	Choledochal Cyst	100	99	99	100	100	100

## Discussion

A slight female preponderance, with a female-to-male ratio of 1.1:1, was noted in patients with obstructive jaundice. Patients aged 51 to 60 years were the most numerous, followed by those aged 41 to 50 years. Our results show similarity with the study conducted by Upadhyaya et al. [5]. The majority of the patients (51.67%) had gallstones.

Diagnostic performance of USG in benign lesions

In the majority of patients, no cause of obstruction could be determined. The correct cause of obstruction was found in nearly one-fifth of the patients. The most common cause of benign biliary obstruction detected on USG was choledocholithiasis, which was similar to studies conducted by Upadhyaya et al. [5], Singh et al. [6], and Siva et al. [7]. The diagnostic performance of USG in choledocholithiasis is relatively poor (one in five cases), though an estimate about the level of obstruction could be made. Our study showed marked similarity with the study conducted by Alsaigh et al. [8], who found that there was 26.6% sensitivity and 100% specificity in detecting CBD stones.

Mirizzi syndrome was one of the most common cases that were misdiagnosed as choledocholithiasis. The diagnostic performance of the USG in benign biliary stricture, ampullary stenosis, periampullary diverticulum, and Mirizzi syndrome was fairly low. Our study related to benign CBD stricture is in accordance with a study conducted by Kaur et al. [9], who found the sensitivity and specificity of USG in diagnosing benign strictures to be 20% and 100%, respectively. The excellent specificity was due to the ability of USG to find true negatives in benign stenosis, thereby demonstrating the origin of the obstruction as a CBD stone or malignant stricture. The low sensitivity numbers were due to the inherent limitations of the approach, which, while displaying indirect indicators of stenosis, did not provide excellent viewing of the distal CBD and the periampullary region, where benign stenosis was frequently situated [9].

A high specificity of USG in detecting benign lesions was found in studies conducted by Prusty et al. (100%) [10], Verma et al. (88.4%) [11], and Ferrari et al. (94%) [12], which is similar to our study.

Only in about half of the patients with benign obstructive lesions could a diagnostically accurate estimate of the level of obstruction be made using USG in our study. Other studies have reported a variation of 27-95% in detecting level and 18-85% in detecting the cause of obstruction through USG [13-17].

Diagnostic performance of USG in malignant lesions

**Figure 12:**
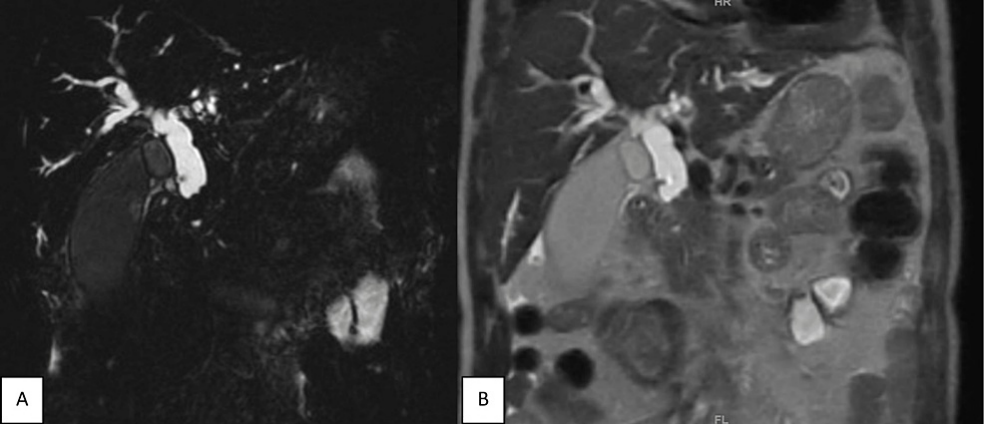
Distal cholangiocarcinoma. Coronal three-dimensional reconstructed image (A) and coronal T2 SSFSE image (B) show shouldering of distal common bile duct mass with dilated proximal biliary system SSFSE: single-shot fast spin echo

In the majority (more than two-thirds) of the patients, a correct diagnosis about the cause of obstruction could be made. The most common malignant cause of biliary obstruction detected on USG was gallbladder carcinoma, followed by cholangiocarcinoma (Figure 12). Diagnostic performance of USG in malignant lesions like gallbladder carcinoma and cholangiocarcinoma was fairly high, which is similar to that of Kaur et al. [9] and Singh et al. [6]. The overall sensitivity, specificity, and accuracy were 66.67%, 100%, and 96%, respectively, for cases with cholangiocarcinoma on USG in the study conducted by Singh et al. [6], which is similar to our study. Hann et al. [18] also found a sensitivity of 87% in detecting Hilar cholangiocarcinoma, which is in concordance with our study.

High sensitivity and specificity of USG in detecting neoplastic lesions were found in studies conducted by Verma et al. (88.4% and 85.3%) [11], Ferrari et al. (61.12% and 98.3%) [12], and Singh et al. (79.17% and 96.15%) [6].

Relatively poor diagnostic performance is noted in periampullary carcinoma and pancreatic carcinoma, likely due to obscuration of the field of view by overlying bowel gas. In patients with malignant obstructive lesions, a fairly high (nine-tenths) diagnostically accurate estimate of the site of obstruction could be made. 

Diagnostic performance of MRCP in benign lesions

**Figure 13:**
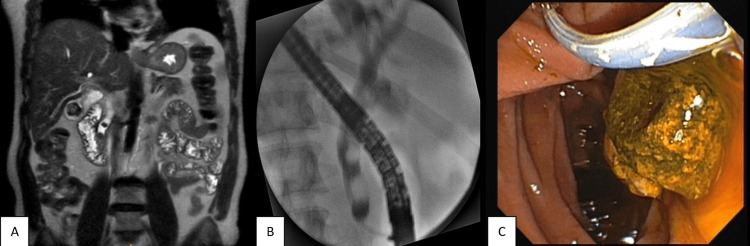
Choledocholithiasis. (A) Coronal T2 SSFSE showing two round calculi in distal common bile duct along with cholelithiasis; (B) ERCP film showing two oval filing defects; (C) Calculi extraction during ERCP ERCP: endoscopic retrograde cholangiopancreatography: SSFSE: single-shot fast spin echo

The majority of patients had a correct diagnosis of the cause of obstruction. The most common benign cause of biliary obstruction detected on MRCP was choledocholithiasis (Figure 13), followed by benign biliary stricture and Mirizzi syndrome. The present study is similar to that of Soto et al., who reported sensitivity and specificity of 94% and 100%, respectively, for detecting biliary calculi in MRCP [19]. Pavone et al. discovered that identifying CBD calculus on MRCP had an 88.9% sensitivity and a 100% specificity [20]. Our study also showed similarity with Guibaud et al., who found 100% accuracy in detecting CBD calculi on MRCP in cases with equivocal sonographic results [21]. Varghese et al. reported a sensitivity of 91%, specificity of 98%, and diagnostic accuracy of 97% on the MRCP [22]. Sugiyama et al. reported a sensitivity of 91%, specificity of 100%, and diagnostic accuracy of 97% on the MRCP [23].

Excellent diagnostic performance was noted in detecting all benign lesions, especially benign CBD strictures. According to a study by Bhatt et al., benign or malignant bile duct strictures and neoplastic lesions in the lower part of the CBD can be examined more effectively by MRCP [24], which is similar to our study. Our study is in concordance with a study conducted by Al-Obaidi et al., in which the sensitivity, specificity, and accuracy of MRCP for cases with benign biliary strictures were 100%, 98.5%, and 98.7%, respectively [25]. Our study is also in concordance with a study conducted by Singh et al., who found 100% accuracy for MRCP in diagnosing benign CBD strictures [6]. Munir et al. found the sensitivity and specificity of MRCP in detecting benign main bile duct strictures to be 83.3% and 97.6%, respectively [26]. Our research is consistent with that of Singh et al., who discovered that MRCP has 100% sensitivity and 98% diagnostic accuracy for benign diseases [6]. MRCP showed excellent diagnostic accuracy in predicting the site of the lesion. The most common level of obstruction was found to be distal CBD.

Diagnostic performance of MRCP in malignant lesions

**Figure 14:**
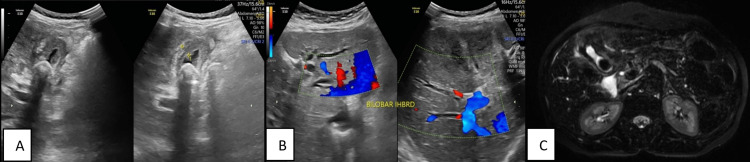
GB carcinoma. (A) Transabdominal USG shows irregularly thickened GB wall with cholelithiasis; (B) Transabdominal USG shows bilobar intrahepatic biliary dilatation; (C) Axial three-dimensional reconstructed image shows irregularly thickened GB wall with surrounding T2 hyperintensity and cholelithiasis GB: gallbladder

In the majority of patients, a correct diagnosis of the cause of obstruction was made through MRCP. The most common malignant cause of biliary obstruction detected on MRCP was gallbladder carcinoma (Figure 14), followed by cholangiocarcinoma. Excellent diagnostic performance was noted in detecting all malignant lesions.

The sensitivity and specificity of MRCP for malignant pathologies found in studies conducted by Munir et al. (92% and 100%) [26], Prusty et al. (93.75% and 100%) [10], Verma et al. (86% and 92%) [11], and Ferrari et al. (90% and 94%) [12] are similar to our study.

The most common site of obstruction on MRCP was the proximal common duct. In our study, MRCP demonstrated excellent diagnostic performance in detecting the site of a lesion, which is consistent with the findings of Singh et al. [6].

Overall diagnostic performance of USG and MRCP

USG was correctly able to predict the cause of obstruction in one-third of all patients. The overall diagnostic accuracy of USG in predicting the site of obstruction was 64.3%. Other studies have reported a variation of 27-95% in detecting levels and 18-85% in detecting the cause of obstruction through USG [21-25]. Our study's overall specificity is comparable to that of Kaur et al. (100%) [9], Shadan et al. (83.3%) [27], and Kurian et al. (97.14%) [28].

MRCP was correctly able to predict the cause of obstruction in the majority (>90%) of patients. The overall diagnostic accuracy of MRCP in predicting the site of obstruction was 98.33%. Our study is in accordance with a study conducted by Farid et al., who found 97% accuracy in detecting the cause and 100% accuracy in detecting the site of obstructive jaundice [29]. Similar studies were conducted by Varghese et al. (sensitivity: 97%) [17], Romagnuolo et al. (sensitivity: 97%) [30], Shadan et al. (sensitivity: 97.7% and specificity: 100%) [27], and Kurian et al. (sensitivity: 97.14%) [28]. Upadhyaya et al. found MRCP was able to detect the level of obstruction in 95.45% of cases and the cause in 87.50% of cases [5]. Other studies have also reported very good results, with the ability to detect levels ranging from 85% to 100% [3,31-33].

On USG, no cause of biliary obstruction was found in nearly half of the patients in whom MRCP showed excellent diagnostic performance.

## Conclusions

The diagnostic performance of USG in benign lesions is low as compared to MRCP. The diagnostic accuracy of USG in predicting the site of obstruction in benign cases is somewhat equivocal. As a result, USG cannot be used as the primary investigation in benign obstructive pathologies. The diagnostic performance of USG in acalculous pathologies is relatively higher as compared to choledocholithiasis. When compared to benign lesions, the diagnostic performance of USG in malignant lesions is slightly higher in detecting the cause and level of obstruction, though less than that of MRCP. In cases of clinical suspicion of a malignant cause of obstructive jaundice, MRCP could be considered the first choice of investigation for evaluation of the nature and level of obstruction. It could be used as the modality of choice and next step of investigation when clinical suspicion of obstructive jaundice is present, and no definite cause of biliary obstruction is seen on USG.

Overall, USG could be used as the initial screening modality of choice for predicting the level and nature of obstruction in patients with a clinical suspicion of obstructive jaundice. MRCP should be the radiological investigation of choice in patients with a clinical suspicion of obstructive jaundice and has the potential to become the new "Gold standard" investigation for diagnosis in patients with biliary obstruction owing to its excellent diagnostic performance and non-invasiveness.
